# Isolation, Identification and Characterization of Yeasts from Fermented Goat Milk of the Yaghnob Valley in Tajikistan

**DOI:** 10.3389/fmicb.2016.01690

**Published:** 2016-11-03

**Authors:** Linnea A. Qvirist, Carlotta De Filippo, Francesco Strati, Irene Stefanini, Maddalena Sordo, Thomas Andlid, Giovanna E. Felis, Paola Mattarelli, Duccio Cavalieri

**Affiliations:** ^1^Department of Biology and Biological Engineering, Chalmers University of TechnologyGothenburg, Sweden; ^2^Institute of Biometeorology, National Research CouncilFlorence, Italy; ^3^Department of Computational Biology, Edmund Mach FoundationSan Michele all'Adige, Italy; ^4^Department of Biotechnology, University of VeronaVerona, Italy; ^5^Department of Agricultural Sciences, University of BolognaBologna, Italy; ^6^Department of Biology, University of FlorenceFlorence, Italy

**Keywords:** yeast, fermented goat milk, Yaghnob Valley Tajikistan, identification, phenotyping, genotyping

## Abstract

The geographically isolated region of the Yaghnob Valley, Tajikistan, has allowed its inhabitants to maintain a unique culture and lifestyle. Their fermented goat milk constitutes one of the staple foods for the Yaghnob population, and is produced by backslopping, i.e., using the previous fermentation batch to inoculate the new one. This study addresses the yeast composition of the fermented milk, assessing genotypic, and phenotypic properties. The 52 isolates included in this study revealed small species diversity, belonging to *Kluyveromyces marxianus, Pichia fermentans, Saccharomyces cerevisiae*, and one *Kazachstania unispora*. The *K. marxianus* strains showed two different genotypes, one of which never described previously. The two genetically different groups also differed significantly in several phenotypic characteristics, such as tolerance toward high temperatures, low pH, and presence of acid. Microsatellite analysis of the *S. cerevisiae* strains from this study, compared to 350 previously described strains, attributed the Yaghnobi *S. cerevisiae* to two different ancestry origins, both distinct from the wine and beer strains, and similar to strains isolated from human and insects feces, suggesting a peculiar origin of these strains, and the existence of a gut reservoir for *S. cerevisiae*. Our work constitutes a foundation for strain selection for future applications as starter cultures in food fermentations. This work is the first ever on yeast diversity from fermented milk of the previously unexplored area of the Yaghnob Valley.

## Introduction

The history of fermented beverages and dairies dates back to more than 3500 years (Cavalieri et al., 2003) and possibly occurred with the first neolitic settlements, fermentation likely evolved to preserve crops and dairies as fermented foods, by creating an environment less favorable for spoilage microorganisms. In many rural areas, spontaneous food fermentations are still the main method for food processing, often using back-slopping to inoculate the new batch by transferring an aliquot of the previous food batch. This method allows for microbial adaptation and natural selection of strains thriving in the food matrix. There are several players involved in spontaneous fermentations, and previous studies have reported isolation of various yeasts and/or bacteria from natural fermentations of e.g., cereal based foods (Hellström et al., 2010; Ogunremi et al., 2015; Todorov and Holzapfel, 2015), or from various milk (Gadaga et al., 2001; Mathara et al., 2004; Bai et al., 2010; Yun Li and Guoqing, 2015), or cheese (Fasoli et al., 2015) fermentations. The analyses of the microbiota associated to spontaneous fermentations allows the isolation of microorganisms possessing properties desirable for implementation in industrial food or feed processes. Furthermore, the microbiota of a traditional food fermentation will likely also reflect the microbiota of the geographical area where it has been produced, as there is a continuous transfer of microbes between the close-by environment and the food fermentation. Those natural fermentations are conducted without pasteurization/sterilization of the substrate, and without applying particular hygienic protocols. Thus, selection of the environmental microbial population may occur only through the fermentative process, by chemico-physical modifications of the substrate induced by microbes themselves.

Both yeasts and bacteria are frequently isolated from fermentations (Tamang et al., 2016) and can possess traits that gives beneficial effects on the food product itself and for the consumer. Probiotic bacteria have been long studied, and lately also commercialized, as health promoting food ingredients, for example in some brands of yogurt (Sen et al., 2002). Recently the use of yeasts as probiotic agents in food has received increased attention. One example is the lactic yeast species *Kluyveromyces marxianus*, frequently isolated from dairy food fermentations. The strain *K. marxianus* B0399^®;^, for example, was shown to have probiotic properties such as the modulation of the immune response in CaCo-2 cell line (Maccaferri et al., 2012) and further showed a positive effect on patients with irritable bowel syndrome (IBS) (Lisotti et al., 2013). Other studies on yeast strains with probiotic properties have investigated their lipolytic and proteolytic properties (Psomas et al., 2001) and the positive effects on the expression of pro-inflammatory cytokine IL-1α (van der Aa Kühle et al., 2005), as well as production of several vitamins, bioactive peptides, and more (Czerucka et al., 2007; Fernandez et al., 2015). Other beneficial effects of introducing selected yeast strains in food processes are for example the ability of such strains to metabolize lactose as a way of producing low lactose dairy products for lactose intolerant consumers (Gadaga et al., 2001; Mathara et al., 2004; Bai et al., 2010; Yun Li and Guoqing, 2015) and yeast strains acting as antagonists toward spoilage or pathogenic microorganisms (Mufandaedza et al., 2006) to mention a few examples. However, for a microorganism to be considered as a probiotic, the ability to survive/pass through the harsh conditions of the gastrointestinal tract (low pH), in presence of ox bile and at a temperature of 37°C with maintained viability is often applied as a first assessment.

The fermented milk of the Yaghnob Valley represents a precious resource for studying spontaneous fermentations for several reasons. First of all, it is one of the few still untapped traditional fermented productions yet to be investigated, hence both the yeast community and their phenotypic properties are unknown. As the use of health promoting microorganisms is of increasing interest, isolation, and phenotyping of strains from a previously unexplored fermented food may yield fruitful information of potentially new probiotic strains for future application in food industries. Further, isolation and identification of yeasts from this geographically unexplored area will add information to the body of knowledge on yeast species distribution and prevalence, and also about the genetic variations of strains evolved in an isolated area such as the Yaghnob Valley. The Yaghnob people are a Tajikistan ethnic minority living through their natural economy in areas remote from the “modern civilization” and avoiding exchanges with it. The long lasting isolation of this population has largely prevented mixing with other populations, thus preventing at the same time the eventual contamination of microbes among fermentative processes. This cultural-economic settings have thus prevented the flux of microorganisms supposed to have homogenized the worldwide populations of some fermentative microbes (Fay and Benavides, 2005).

The aim of this work was to investigate the yeast biodiversity of the Yaghnob populations traditionally fermented goat milk and to perform genotypic and phenotypic characterization of the isolated yeasts in order to contribute to the body of knowledge of yeasts in traditional food fermentations, and to add new information for a previously unexplored geographical area.

## Materials and methods

### Yeast isolation

From original Yaghnob yogurt, isolations were done on different common agar lab media under aerobic conditions and at 30°C. Colonies were firstly selected based on colony morphology, aiming at selecting colonies of varying morphology, and thereafter additional colonies were randomly selected. Five isolates were obtained from M17 medium (annotated AL 1-5), seven isolates on deMan, Rosa and Sharp (MRS) medium (annotated CL 1-7), 14 isolates from MRS pH 5.4 medium (annotated DL 1-12), 12 isolates on Wallerstein Laboratory (WL) medium (annotated BL3-14), and two isolates on Yeast extract, Peptone, Dextrose (YPD) medium (1% yeast extract, 2% peptone, 2% dextrose) (annotated BL1-2). The yogurt was further maintained in-house by regular backslopping into pasteurized cow milk. Isolation from in-house maintained yogurt was done on YPD agar supplemented with chloramphenicol (100 μg/ml) (YPD+Cam). The original sample had been maintained in-house by repeated backslopping according to the procedure by the Yaghnob population, but using pasteurized cow milk instead of goat milk, for a total time period of 3 years. Twelve colonies of varying morphology were selected from the maintained sample, annotated TJY50-61. Purity was checked by streaking all isolates on YPD agar and pure cultures were maintained on agar of the same medium at 4°C for short term storage, and in YPD broth supplemented with glycerol (15% v/v) at −80°C for long term storage.

### Genotypic characterization

#### ITS1-4 sequencing

Yeast genomic DNA was extracted from isolated colonies as previously described (Hoffman and Winston, 1987). Strains were identified by amplification and sequencing of the ribosomal Internal Transcribed Spacer (ITS) region, using ITS1 (5′-GTTTCCGTAGGTGAACTTGC-3′) and ITS4 (5′-TCCTCCGCTTATTGATATGC-3′) primers, as previously described (Sebastiani et al., 2002). Species attribution was obtained by using the Basic Logarithmic Alignment Search Tool (BLAST) algorithm in the National Centre for Biotechnology Information (NCBI) database (minimum 97% sequence similarity and 95% coverage). All ITS1-4 sequences were submitted to GenBank and the accession numbers are presented in Table 1. Multiple alignments were performed using online tool ClustalW2.

**Table 1:** **The 52 isolates and their respectively species identity, isolation medium, fermentation origin, and the GenBank accession number is indicated in the table**.

**Isolate**	**Species**	**Isolation medium**	**Fermentation sample**	**GenBank accession number**
AL1	*Kluyveromyces marxianus*	M17	Original	KX905245
AL2	*Kluyveromyces marxianus*	M17	Original	KX905246
AL3	*Kluyveromyces marxianus*	M17	Original	KX905247
AL4	*Kluyveromyces marxianus*	M17	Original	KX905248
AL5	*Kluyveromyces marxianus*	M17	Original	KX905249
BL1	*Kluyveromyces marxianus*	YPD	Original	KX905250
BL3	*Kluyveromyces marxianus*	WL	Original	KX905251
BL4	*Kluyveromyces marxianus*	WL	Original	KX905252
BL5	*Kluyveromyces marxianus*	WL	Original	KX905253
BL6	*Kluyveromyces marxianus*	WL	Original	KX905254
BL7	*Kluyveromyces marxianus*	WL	Original	KX905255
BL8	*Kluyveromyces marxianus*	WL	Original	KX905256
BL12	*Kluyveromyces marxianus*	WL	Original	KX905257
BL13	*Kluyveromyces marxianus*	WL	Original	KX905258
BL14	*Kluyveromyces marxianus*	WL	Original	KX905259
CL5	*Kluyveromyces marxianus*	MRS	Original	KX905260
CL6	*Kluyveromyces marxianus*	MRS	Original	KX905261
DL2	*Kluyveromyces marxianus*	MRS pH 5.4	Original	KX905262
DL4	*Kluyveromyces marxianus*	MRS pH 5.4	Original	KX905263
DL5	*Kluyveromyces marxianus*	MRS pH 5.4	Original	KX905264
DL6	*Kluyveromyces marxianus*	MRS pH 5.4	Original	KX905265
DL10a	*Kluyveromyces marxianus*	MRS pH 5.4	Original	KX905266
DL10b	*Kluyveromyces marxianus*	MRS pH 5.4	Original	KX905267
DL11	*Kluyveromyces marxianus*	MRS pH 5.4	Original	KX905268
DL12	*Kluyveromyces marxianus*	MRS pH 5.4	Original	KX905269
TJY52	*Kluyveromyces marxianus*	YPD+Cam	Maintained	KX905270
TJY54	*Kluyveromyces marxianus*	YPD+Cam	Maintained	KX905271
TJY59	*Kluyveromyces marxianus*	YPD+Cam	Maintained	KX905272
TJY60	*Kluyveromyces marxianus*	YPD+Cam	Maintained	KX905273
BL9	*Saccharomyces cerevisiae*	WL	Original	KX905274
BL10	*Saccharomyces cerevisiae*	WL	Original	KX905275
BL11	*Saccharomyces cerevisiae*	WL	Original	KX905276
CL2	*Saccharomyces cerevisiae*	MRS	Original	KX905277
CL3	*Saccharomyces cerevisiae*	MRS	Original	KX905278
CL4	*Saccharomyces cerevisiae*	MRS	Original	KX905279
DL3	*Saccharomyces cerevisiae*	MRS pH 5.4	Original	KX905280
DL7	*Saccharomyces cerevisiae*	MRS pH 5.4	Original	KX905281
TJY58	*Saccharomyces cerevisiae*	YPD+Cam	Maintained	KX905282
TJY61	*Saccharomyces cerevisiae*	YPD+Cam	Maintained	KX905283
BL2	*Pichia fermentans*	YPD	Original	KX905284
CL1	*Pichia fermentans*	MRS	Original	KX905285
CL7	*Pichia fermentans*	MRS	Original	KX905286
DL1	*Pichia fermentans*	MRS pH 5.4	Original	KX905287
DL8a	*Pichia fermentans*	MRS pH 5.4	Original	KX905288
DL8b	*Pichia fermentans*	MRS pH 5.4	Original	KX905289
DL9	*Pichia fermentans*	MRS pH 5.4	Original	KX905290
TJY50	*Pichia fermentans*	YPD+Cam	Maintained	KX905291
TJY53	*Pichia fermentans*	YPD+Cam	Maintained	KX905292
TJY55	*Pichia fermentans*	YPD+Cam	Maintained	KX905293
TJY56	*Pichia fermentans*	YPD+Cam	Maintained	KX905294
TJY57	*Pichia fermentans*	YPD+Cam	Maintained	KX905295
TJY51	*Kazachstania unispora*	YPD+Cam	Maintained	KX905296

#### PCR-RFLP analysis

Restriction fragment length polymorphism (RFLP) analyses of the amplified ITS1-4 region were performed as described by Esteve-Zarzoso et al. (1999), using *HaeIII* or *HinfI*.

#### Microsatellite analysis

In this work, microsatellite analysis was performed only for *Saccharomyces cerevisiae* isolates. The genomic DNA was extracted by phenol-chloroform-isoamyl alcohol method to be used for (GTG)_5_ Rep PCR. The PCR mixture consisted of 1.25 μL buffer (10x), 1 μL MgCl_2_ (25 mM), 2.5 μL dNTP (5 mM), 0.4 μL forward primers (10 mM), 0.4 μL reverse primer (10 mM), 0.05 μL AmpliTaq Gold® DNA polymerase (5 U/μL), 4.4 μL H_2_O and 2.5 μL DNA template (10 ng/μL). The investigated loci were C3, C4, C5, C6, C8, C11, SCYOR267c, YKL172w, SCAAT1, SCAAT3, SCAAT5, and YPL3 (Legras et al., 2005). The PCR program consisted of an initial step at 95°C for 5 min, followed by 35 cycles of 95°C for 0.5 min, 57°C for 2 min, and 72°C for 1 min, before a final elongation step at 60°C for 30 min. Thereafter samples were cooled down to 8°C until further use. The PCR products were checked by gel electrophoresis. The chord distances (Dc) were calculated among each couple of strains with a laboratory-made R script. The phylogenetic tree was obtained from the distance matrices with the Phylip Neighbor 3.67 package and drawn up using Figtree. The tree was rooted using the midpoint method.

Strains ancestry was estimated by using the model-based program Structure (Pritchard et al., 2000). *K* = 7 was chosen as the most representative of the population structure for the microsatellite sequences. The results of 10 independent Structure chains were combined with CLUMPP (Jakobsson and Rosenberg, 2007).

### Phenotypic characterization

#### Phytate utilization

The strains from the Yaghnob yogurt were screened for their ability to degrade phytate in a nutrient deficient medium, consisting of phytate (3 g/L) and glucose (20 g/L) in succinate buffer at pH 5.5. A volume of 195 μL of the medium was dispensed in each well of a micro plate, and inoculated in duplicate using 5 μL from overnight precultures in YPD. Incubation was done at 30°C with 150 rpm orbital shaking for 48 h. After 48 h of incubation, 22 μL of 5 M HCl was added to each well to stop the phytate degradation. Cells were allowed to sediment, and thereafter 150 μL cell-free sample was mixed with 200 μL of 0.5 M HCl before analyzing the phytate (IP_6_) concentration by High Pressure Ion Chromatography (HPIC). The HPIC analysis method has been previously described by Carlsson et al. (2001).

The isolates were further assessed for their ability to release extracellular non-cell-bound phytase to the surrounding medium. Inoculations were done in 4 mL volumes of Yeast Nitrogen Base plus Yeast Extract (YNB+YE) (6.5 g/L YNB w/o phosphate, 10 g/L yeast extract and 20 g/L glucose in succinate buffer at pH 5.5) to a starting optical density at 600 nm (OD_600_) of about 0.1. The YNB+YE medium has previously shown to trigger release of phytase enzymes to the surrounding medium (Hellström et al., 2015). The incubation was performed for 24 h at 30°C with stirring. After incubation, cells were pelleted by centrifugation at 5000 × g, and the cell-free supernatant was used for assay of phytase activity as previously described (Qvirist et al., 2015). The assay samples were analyzed for IP_6_ concentration using HPIC as previously described (Carlsson et al., 2001), and compared with the phytase positive reference strain *Pichia kudriavzevii* TY13 from previous work (Qvirist et al., 2015).

#### Growth on different carbon sources, pH, temperatures, and ox bile concentrations

To investigate the strains ability to grow on different carbon sources, 6.7 g/L YNB without carbon source (with amino acids) in succinate buffer (pH 5.5) was supplemented with 20 g/L of one of the following carbon sources; glucose, sucrose, lactose, maltose, mannitol, arabinose, xylose, and galactose. The strains were also tested for growth in 8 different media based on 1% yeast extract, 2% peptone; supplemented with either glucose at 50 or 60% (w/v), or ethanol at 1, 6, or 12% or lactic acid at 1, 6, or 12% (v/v). All isolates were also investigated for their ability to grow in YPD broth at different temperatures (4, 27, 37, 40, 42, 46, and 48°C), at different pH (4.8, 3, and 2), and at different levels of added ox bile (0.5, 1, and 2% v/v).

Cultures were done for each strain by adding 5 μL preculture (from overnight incubation in YPD) into 195 μL of the experimental media, giving a starting OD (630 nm) between 0.08–0.1. Incubations were done at 150 rpm orbital shaking for 3 days at 30°C for all tests except the pH and ox bile tests which were done at 37°C. For strain TJY51, 27°C was used due to its poor growth at higher temperatures. The optical density was read at 630 nm, and values below 0.2 are considered as negative, from 0.2 to 0.4 as positive but inhibited growth and above 0.4 as positive growth.

Further, the viability of strains after incubation at (i) 48°C in YPD broth for 24 h, and (ii) in YPD broth of pH 2 for 2 h at 30°C was investigated. To assess the viability, 10 μL of the cell suspensions were spotted in duplicates onto YPD agar, together with a negative control from cultivation in normal YPD at 30°C. The YPD agar plates were incubated at 30°C overnight and then visual evaluation of the growth was done.

All tests were conducted in triplicates.

#### Invasiveness of isolates

All isolates were investigated for invasiveness on YPD agar in triplicates. Volumes of 2.5 μL liquid yeast suspensions were spotted onto the surface of YPD agar and incubated at 27°C for 5 days. Thereafter cells were removed and plates were carefully washed with deionized water before being stained as described by Vopálenská et al. (2005). The invasiveness was graded from 0 (not invasive) to 4 (highly invasive).

#### Resistance toward oxidative stress

All isolates were investigated for resistance toward oxidative stress. Cell suspensions from each strain was spread on YPD agar and allowed to absorb, thereafter a paper disk soaked in hydrogen peroxide (H_2_O_2_) was placed in the center of the agar plate. The resistance toward the oxidative stress was determined by measuring the radius from the border of the growing yeast to the H_2_O_2_-disk after 2 days of incubation at 27°C.

#### Hyphae formation

To investigate the isolates ability to produce hyphae and pseudo hyphae, 5 μL preculture was inoculated into 195 μL of YPD, YNB (without carbon source or ammonium sulfate) and RPMI (Roswell Memorial Park Institute) media, and incubated at 27 and 37°C (only 37°C was used for RPMI) for a total of 7 days, with microscopic investigation at 2 and 7 days.

#### Antifungal tolerance

The antifungal tests were carried out according to the Eucast protocol (Eucast, 2012) with minor adaptations. Selected strains were cultivated in YPD based medium containing the antifungals fluconazole (32–128 mg/L), clotrimazole (0.06–0.5 mg/L) or amphotericin B (0.06–0.5 mg/L) respectively to determine their minimum inhibitory concentration (MIC). The strains used were *Pichia fermentans* CL1 and BL2, *Kluyveromyces marxianus* BL3, BL8, DL4, and TJY52, *S. cerevisiae* CL2 and BL9, and the *Kazachstania unispora* TJY51. Precultures were prepared overnight and the biomass was then washed and resuspended in sterile saline before inoculation into a final volume of 200 μL in the test plates, yielding a starting concentration of about 0.5–2.5^*^10^5^ CFU/mL. Positive controls were made by inoculation into YPD without antifungal drug, and negative controls were made by using the test media without inoculation. Incubations were done in duplicates at 30°C with 170 rpm in micro well plates. After 24 h of incubation, microbial growth was evaluated by optical density at 530 nm by using a spectrophotometer (Multiskan EX, Thermo Scientific). The MIC was defined as the lowest concentration in absence of visible growth and confirmed by OD analysis. OD data above 0.2 was considered as positive growth, while for wells having growth below OD 0.2, re-inoculation was done and the plates were incubated for another 24 h to ensure the result as true negative.

### Statistical analyses

The growth data from the phenotypic characterizations were subjected to statistical evaluation. For each strain, the mean value of duplicate cultures were used. Principal component analysis (PCA) was performed on the OD measurement after standardization (zero mean, unit deviation), and permANOVA (using the vegan R package Jari Oksanen et al., 2015) for statistical analysis. For the two genotypes within the *K. marxianus* species, Wilcoxon rank-sum tests were performed using the stats R package (version 3.1.2).

## Results

### Yeast strain identification

A total of 52 strains were isolated from either original (40 isolates) or maintained (12 isolates) Yaghnob yoghurt. The isolated yeasts belonged to the species *Kluyveromyces marxianus* (29 isolates), *S. cerevisiae* (10 isolates), *P. fermentans* (12 isolates), and *K. unispora* (1 isolate) (Table 1). Strain characterization was firstly assessed by PCR-RFLP analysis after digestion of the amplified ITS1-4 region using the enzymes *HinfI* or *HaeIII* (Table 2).

**Table 2:** **Sizes in base pairs (bp) of PCR products from alpifications fo the ITS1-4 region after restriction digestion using enzymes *Hae*III and *Hin*fI respectively for each strain**.

**Species**	**Strain**	**Restriction fragments (bp)**^**a**^	
		***Hae*III**	***Hin*fI**
*Kluyveromyces marxianus*	AL1	655, 80	240, 185, 140, 80
*Kluyveromyces marxianus*	AL2	655, 80	240, 185, 120, 80
*Kluyveromyces marxianus*	AL3	655, 80	240, 185, 120, 80
*Kluyveromyces marxianus*	AL4	655, 80	240, 185, 120, 80
*Kluyveromyces marxianus*	AL5	655, 80	240, 185, 140, 80
*Kluyveromyces marxianus*	BL1	655, 80	240, 185, 120, 80
*Kluyveromyces marxianus*	BL3	655, 80	240, 185, 120, 80
*Kluyveromyces marxianus*	BL4	655, 80	240, 185, 140, 80
*Kluyveromyces marxianus*	BL5	655, 80	240, 185, 120, 80
*Kluyveromyces marxianus*	BL6	655, 80	240, 185, 140, 80
*Kluyveromyces marxianus*	BL7	655, 80	240, 185, 140, 80
*Kluyveromyces marxianus*	BL8	655, 80	240, 185, 120, 80
*Kluyveromyces marxianus*	BL12	655, 80	240, 185, 140, 80
*Kluyveromyces marxianus*	BL13	655, 80	240, 185, 140, 80
*Kluyveromyces marxianus*	BL14	655, 80	240, 185, 140, 80
*Kluyveromyces marxianus*	CL5	655, 80	240, 185, 120, 80
*Kluyveromyces marxianus*	CL6	655, 80	240, 185, 140, 80
*Kluyveromyces marxianus*	DL2	655, 80	240, 185, 140, 80
*Kluyveromyces marxianus*	DL4	655, 80	240, 185, 140, 80
*Kluyveromyces marxianus*	DL5	655, 80	240, 185, 140, 80
*Kluyveromyces marxianus*	DL6	655, 80	240, 185, 140, 80
*Kluyveromyces marxianus*	DL10a	655, 80	240, 185, 140, 80
*Kluyveromyces marxianus*	DL10b	655, 80	240, 185, 140, 80
*Kluyveromyces marxianus*	DL11	655, 80	240, 185, 140, 80
*Kluyveromyces marxianus*	DL12	655, 80	240, 185, 140, 80
*Kluyveromyces marxianus*	TJY52	655, 80	240, 185, 120, 80
*Kluyveromyces marxianus*	TJY54	655, 80	240, 185, 120, 80
*Kluyveromyces marxianus*	TJY59	655, 80	240, 185, 120, 80
*Kluyveromyces marxianus*	TJY60	655, 80	240, 185, 120, 80
*Saccharomyces cerevisiae*	BL9	320, 230, 180, 150	365, 155
*Saccharomyces cerevisiae*	BL10	320, 230, 180, 150	365, 155
*Saccharomyces cerevisiae*	BL11	320, 230, 180, 150	365, 155
*Saccharomyces cerevisiae*	CL2	320, 230, 180, 150	365, 155
*Saccharomyces cerevisiae*	CL3	320, 230, 180, 150	365, 155
*Saccharomyces cerevisiae*	CL4	320, 230, 180, 150	365, 155
*Saccharomyces cerevisiae*	DL3	320, 230, 180, 150	365, 155
*Saccharomyces cerevisiae*	DL7	320, 230, 180, 150	365, 155
*Saccharomyces cerevisiae*	TJY58	320, 230, 180, 150	365, 155
*Saccharomyces cerevisiae*	TJY61	320, 230, 180, 150	365, 155
*Pichia fermentans*	BL2	340, 80	250, 200
*Pichia fermentans*	CL1	340, 80	250, 200
*Pichia fermentans*	CL7	340, 80	250, 200
*Pichia fermentans*	DL1	340, 80	250, 200
*Pichia fermentans*	DL8a	340, 80	250, 200
*Pichia fermentans*	DL8b	340, 80	250, 200
*Pichia fermentans*	DL9	340, 80	250, 200
*Pichia fermentans*	TJY50	340, 80	250, 200
*Pichia fermentans*	TJY53	340, 80	250, 200
*Pichia fermentans*	TJY55	340, 80	250, 200
*Pichia fermentans*	TJY56	340, 80	250, 200
*Pichia fermentans*	TJY57	340, 80	250, 200
*Kazachstania unispora*	TJY51	550, 150	370

a *Fragments smaller than 80 bp could not be distinguished, but probably bands exists also at 80 and 65 bp for K. marxianus after digestion with HinfI, and at 30 bp for P. fermentans after digestions with HaeIII, as reported by Esteve-Zarzoso et al. (1999)*.

**Figure 1:**
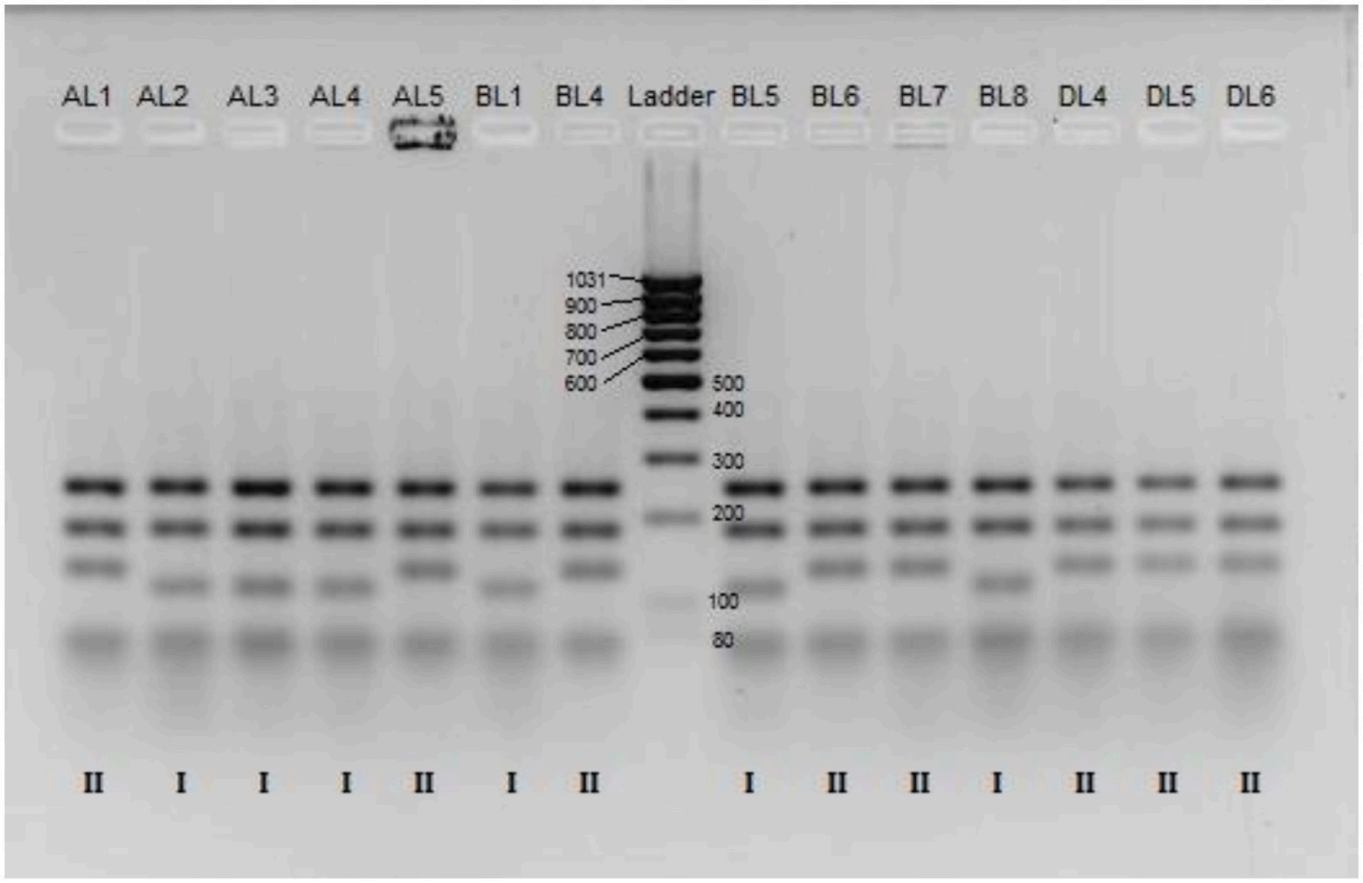
**RFLP patterns for a selected set of *K. marxianus* strains after digestion of the ITS1-4 region by *HinfI* and separation on agarose gel**. The lanes contain, from left to right, samples of strain; AL1, AL2, AL3, AL4, AL5, BL1, BL4, Low Range DNA ladder, BL5, BL6, BL7, BL8, DL4, DL5, and DL6. The genotypic groups, Group I or Group II, is indicated for each strain below each respectively lane.

The PCR-RFLP analysis revealed that within the *K. marxianus* species there are two groups corresponding to different band patterns after digestion with *HinfI* (Figure 1). All the *K. marxianus* isolates have bands length at 240, 185, and 80 bp, but only 12 out of the 29 strains show the frequently reported *K. marxianus* profile (Esteve-Zarzoso et al., 1999; Bockelmann et al., 2008; Pham et al., 2011) with a band at 120 bp (from now on referred to as Group I), while the other 17 strains show a larger band, approximately of 140 bp (from now on referred to Group II).

**Figure 2:**
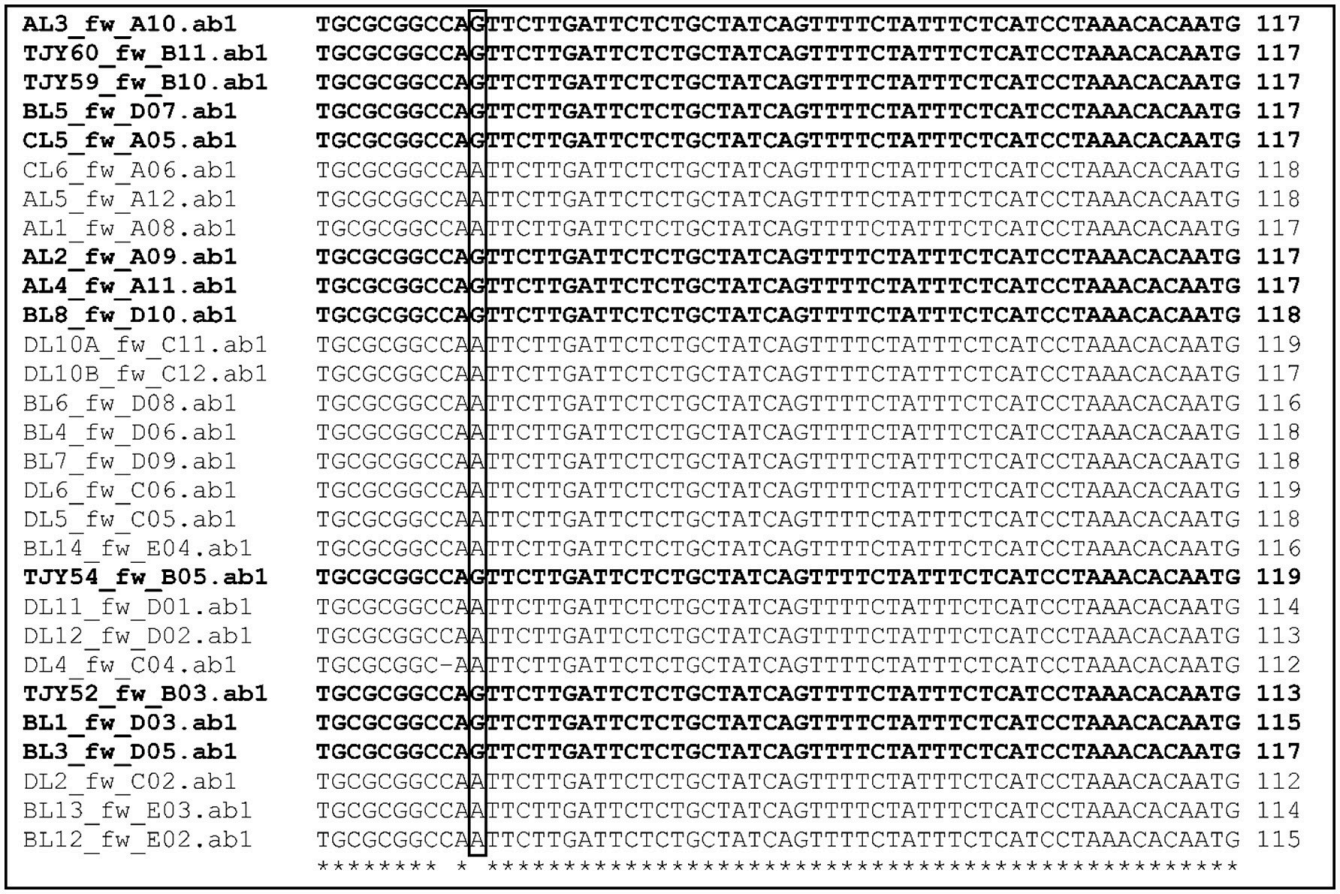
**Multiple sequence alignment of the ITS1-4 sequences from *K. marxianus* strains**. The location of nucleotide variation is indicated by the box. The two groups of *K. marxianus* are marked by bold text (Group I) or normal text (Group II).

To further assess the genetic differences between Group I and Group II of *K. marxianus* isolates, the ITS1-4 sequences were aligned. The alignment revealed that the two groups are separated by having a G (Group I) or an A (Group II) in one of the nucleotide positions marked in Figure 2. To note, all the *K. marxianus* strains isolated in MRS (pH 5.4) medium possess the A allele (8 strains, DL series), whereas the strains isolated on YPD medium both before and after yogurt in-house maintenance bore the G allele (5 strains, TJY series and strain BL1). This indicates that the two *K. marxianus* sub-populations are characterized genetically by two alleles in the ITS1-5.8S-ITS2 region. The combination of genetic and phenotypic differences between the two groups of *K. marxianus* strains may indicate a substantial genomic difference, possibly influencing different phenotypic traits such as tolerance to different environmental (chemico-physical) characteristics.

### Microsatellites

**Figure 3:**
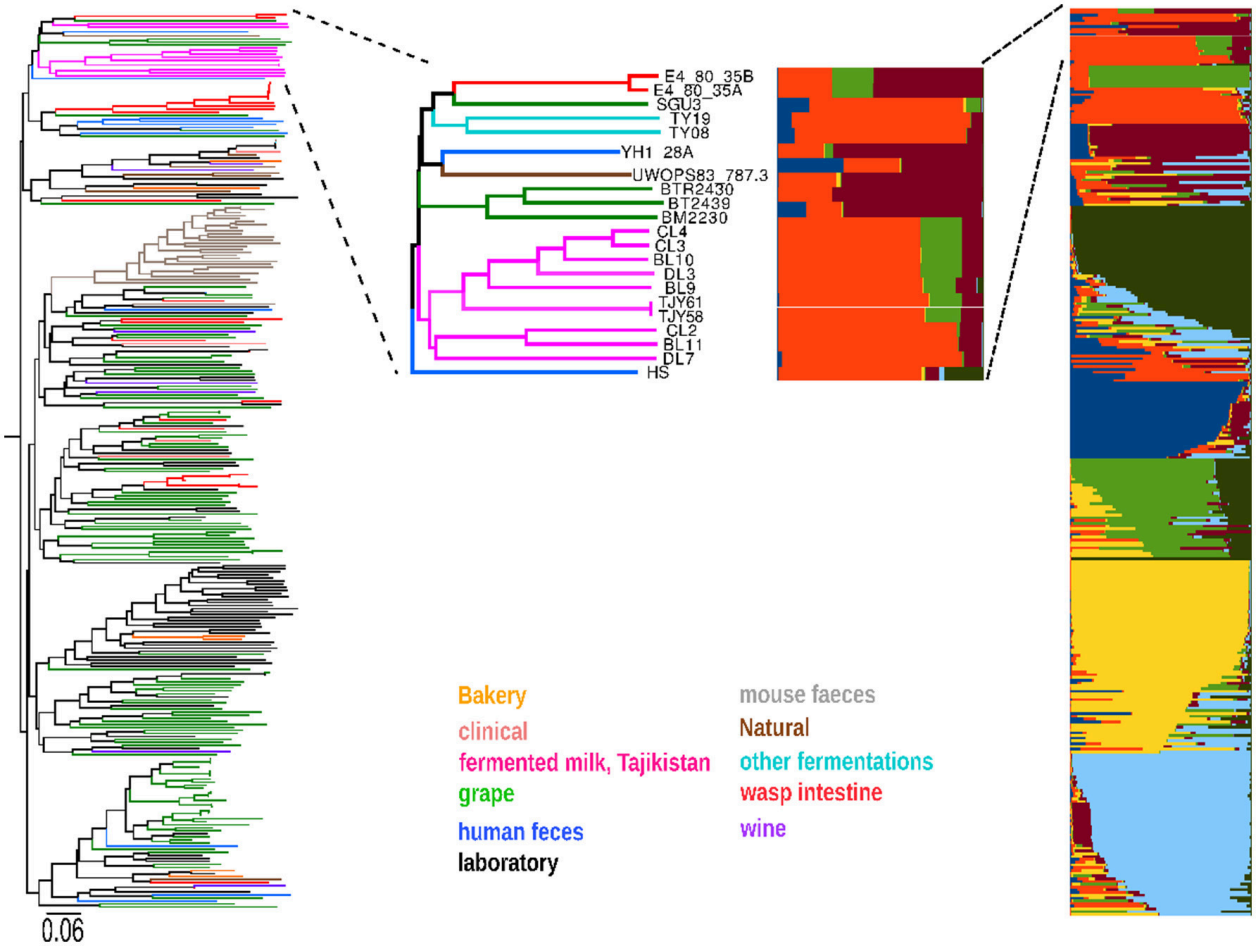
**The ***Saccharomyces cerevisiae*** strains isolated in the Yaghnob yoghurt (in pink color) were compared to a set of around 350 previous ***S. cerevisiae*** isolates from various worldwide origins (left part of figure), revealing that the Yaghnob strains cluster separately and apart from the previous isolates**. The ancestry analysis of those strains (right part of figure) further show that the *S. cerevisiae* from the Yaghnob yoghurt work are all of mosaic ancestry, and constitute two genetically different groups, originating from two ancestors (red and orange) or three ancestors (red, orange, and light green).

Among all the yeast species involved in fermentative processes coupled to food and beverage production, a particular interest has been given to the budding yeast *S. cerevisiae*, known to be the principal player in wine, beer and bread fermentations. We thus analyzed the microsatellite profiles of our *S. cerevisiae* isolates from the Yaghnob fermentation together with the microsatellite data obtained from 350 *S. cerevisiae* strains isolated worldwide from a vast plethora of sources. The phylogenetic analysis (Figure 3) revealed that the *S. cerevisiae* strains found in the Yaghnob yogurt cluster apart from the worldwide strains. The Yaghnob strains clustered close to strains isolated from a wide variety of sources, most interestingly insect intestines (red), human feces (blue), bread fermentations (yellow), and wild sources such as tree barks or soils (brown). It is noteworthy that the Yaghnobi strains appear isolated from the wine strains. In previous studies, several of these strains were shown to have a mosaic genome as a common feature (Legras et al., 2005).

The mosaic nature of the genome of these strains was also confirmed by means of ancestry analysis. The analysis revealed that the *S. cerevisiae* strains isolated from Yaghnob yogurt fell in two groups, both having mosaic ancestry (Figure 3), but originating from different sets of ancestors. Both groups were inferred to descend from a common ancestor (orange), from which directly originated a set of strains isolated from human feces (blue strains, i.e., YP4_40D, YA5-28C). The larger ancestry group contained the strains CL3, CL4, DL3, BL9, BL10, TJY58, and TJY61, originating from an ancestor (red) shared with strains isolated from wild sources, and from a third ancestor (light green) shared with the meiotic segregants of a strain isolated from the intestine of social wasps (F31x). The second ancestry group consisted of the strains CL2, BL11, and DL7 originating from two of the three ancestors inferred for the other group (orange and red).

Furthermore, we did not identify any genotypic differences among the *S. cerevisiae* strains isolated with different isolation media, as we did for the *K. marxianus* strains. This could be ascribed to the fact that the *S. cerevisiae* strains were not affected by the same selective pressures as *K. marxianus*.

### Phenotypic characterization

**Figure 4:**
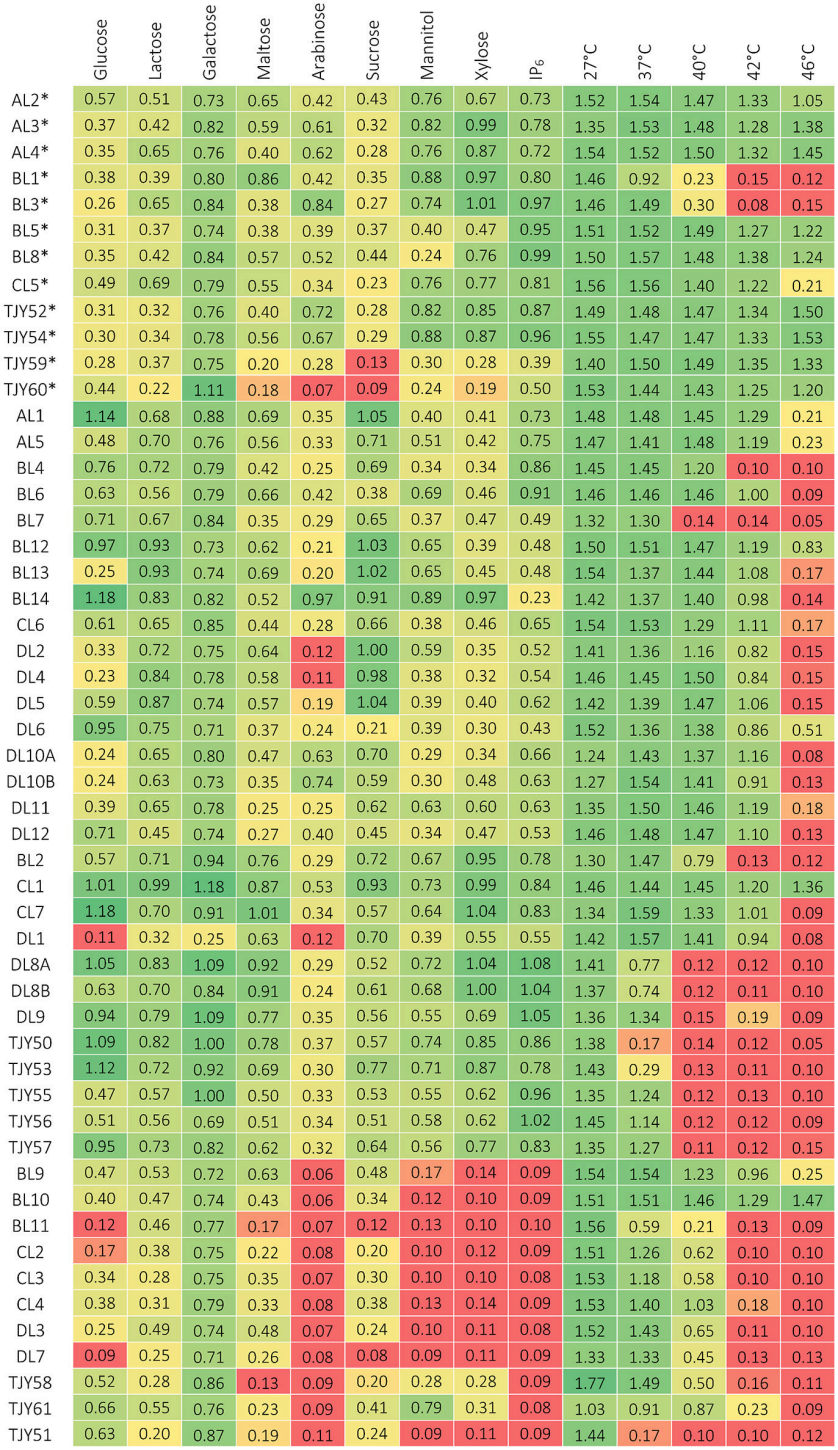
**Growth data after 3 days of incubation, measured as optical density (at 630 nm) for each strain when grown on different carbon sources and at different cultivation temperatures**. Growth below 0.2 is considered as negative (red), growth between about 0.2–0.4 is considered as positive but repressed (yellow) and above circa 0.4 is positive growth (green). ^*^Indicates the *K. marxianus* strains belonging to genotype group I.

**Figure 5:**
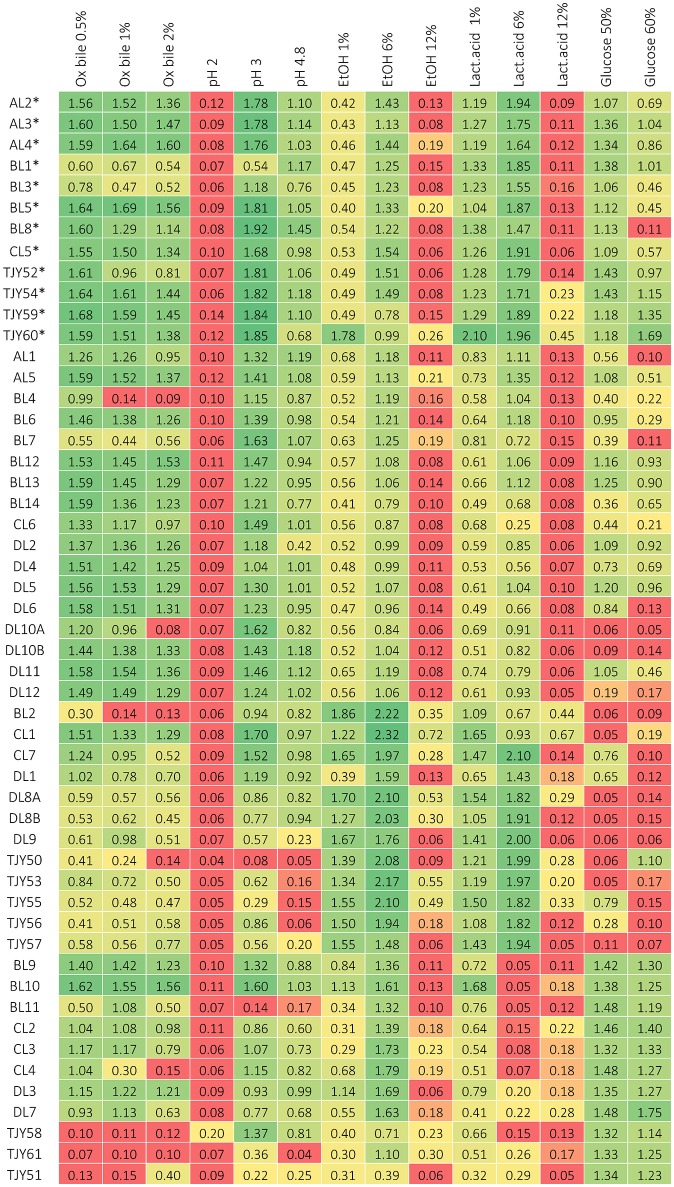
**Growth data after 3 days of incubation, measured as optical density (at 630 nm) for each strain when grown in cultivation media containing either ox bile, ethanol, or lactic acid, or with modified pH, or in high-glucose media to induce osmotic stress respectively**. Growth below 0.2 is considered as negative (red), growth between about 0.2–0.4 is considered as positive but repressed (yellow) and above circa 0.4 is positive growth (green). ^*^Indicates the *K. marxianus* strains belonging to genotype group I.

The results of phenotypic characterizations are shown in Figure 4 for growth in media based on different carbon sources or growth at different cultivation temperatures, and in Figure 5 for growth in presence of ox bile, at low pH, in presence of ethanol or lactic acid and in osmotic stress inducing media.

The *K. marxianus* strains showed remarkably broad substrate utilization and high tolerance to elevated incubation temperatures. Comparison between the two genotype groups I and II were also done and is presented in **Figure 7**.

The *S. cerevisiae* strains grew well up to 37°C, and two strains (BL9 and BL10) grew even at 46°C. All strains except BL11 and TJY61 also grew well at pH 3. Our data show that all strains could utilize glucose, galactose and to some extent also lactose. All strains except BL11 and TJY58 also grew on maltose, and all strains except BL11 and DL7 showed some growth on sucrose. Strains TJY58 and TJY61 seemed able to grow in the mannitol and the xylose based medium as well. All strains could grow in ethanol at 6%, and three strains (DL7, TJY58, and CL2) showed positive but impaired growth at 12% concentration, and only one strain (TJY61) grew well also at 12%. All strains showed high resistance to osmotic stress.

Within the *P. fermentans* species all strains grew well at 37°C, and could utilize all carbon sources tested, with exception of strain DL1 (being negative for arabinose and xylose). Large variations in pH tolerance was observed in this species. The growth in the ethanol and lactic acid media were high within this species, having 8 strains growing at 12% lactic acid and 6 strains growing at 12% ethanol.

The *K. unispora* strain appears rather fastidious and showed to be sensitive to most stresses tested, except for the osmotic stress (induced by 60% glucose) where it together with the genetically close species *S. cerevisiae* show high growth.

It should be pointed out that all strains in this study showed fully recovered growth after incubation at pH 2 for 2 h, which indicates that they could survive through the stomach passage to the intestinal tract.

**Figure 6:**
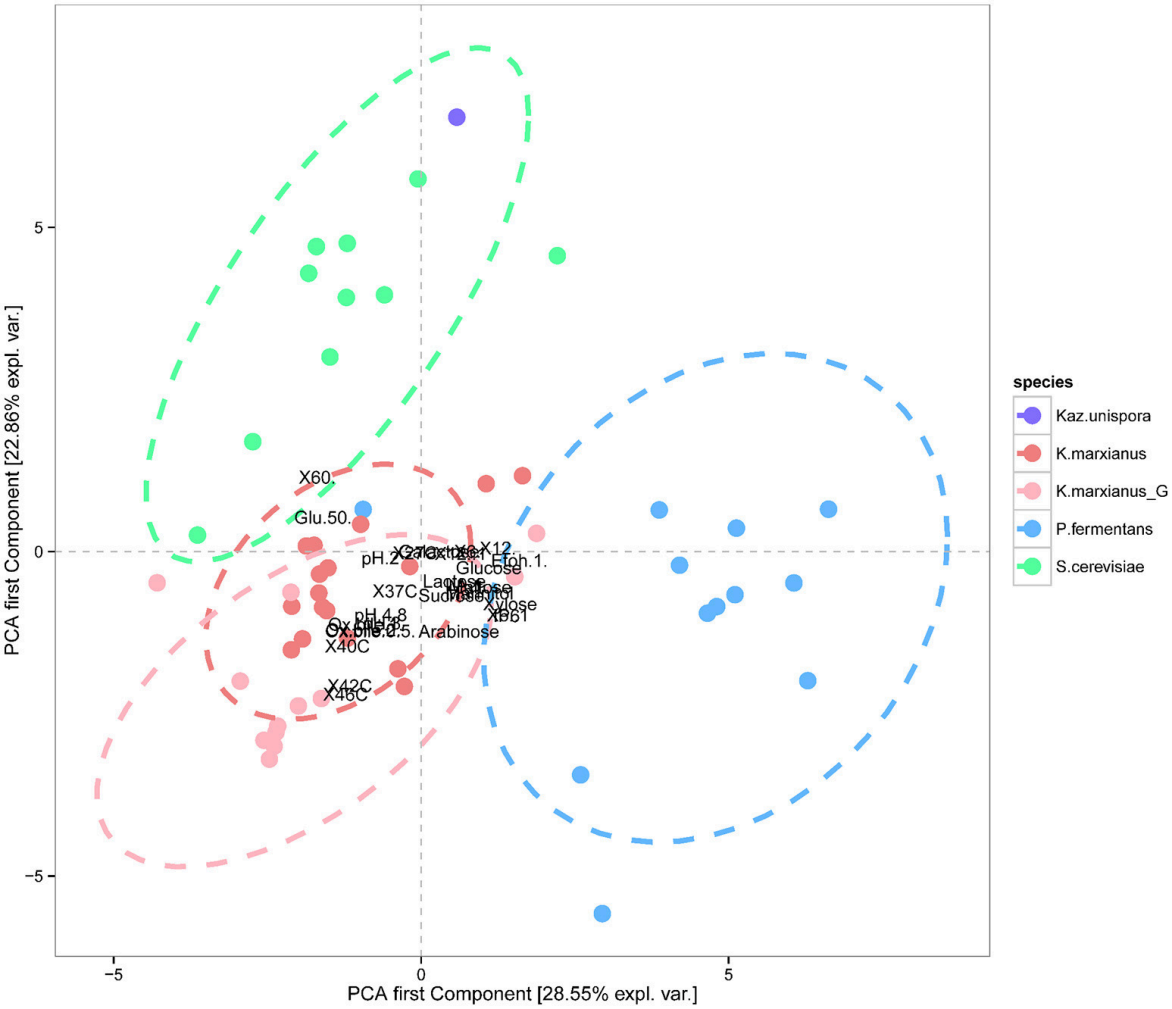
**Principal component analysis (PCA) revealing that the three different species ***S. cerevisiae*** (green), ***P. fermentans*** (blue), and ***K. marxianus*** (pink for Group II and light pink for Group I) could be phenotypically separated from each other (***p*** <0.001), and that the strain of ***Kazachstania unispora*** (purple) cluster together with the ***S. cerevisiae*** strains**. The analysis also showed that the two genetically different groups of *K. marxianus*, Group II (A-nucleotide) in pink and Group I (G-nucleotide) in light pink, could be separated by means of phenotypic characterization. Ellipses were drawn to indicate the data grouping at 95% confidence assuming a multivariate t-distribution of data.

The data obtained from the phenotypic characterizations presented in Figures 4, 5 were further used for a PCA where the isolates belonging to the three species, *K. marxianus, P. fermentans*, and *S. cerevisiae* could be clustered separately (*p* < 0.001), showing also that *K. unispora* phenotypically cluster together with the *S. cerevisiae* strains (Figure 6). In addition, the two genetically different groups within the *K. marxianus* isolates clustered in function of their phenotypic traits, revealing that the two genetically different groups also are phenotypically different.

**Figure 7:**
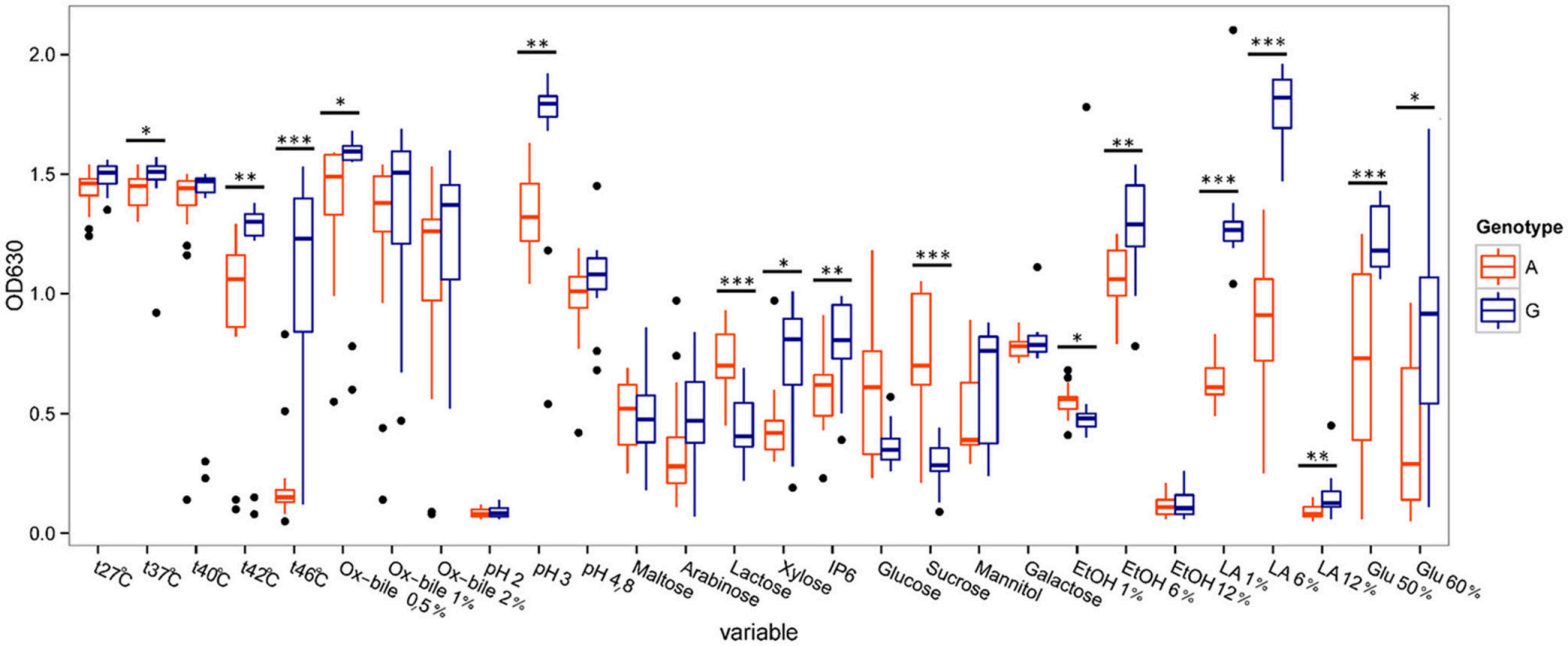
**Comparison of phenotypic characteristics between ***Kluyveromyces marxianus*** genotype Group II (A-nucleotide, orange color) and Group I (G-nucleotide, blue color)**. Significant differences are marked by ^*^(*p* < 0.05), ^**^(*p* < 0.01) or ^***^(*p* < 0.001). Filled dots represent outliers (1.5 times the interquartile range above the upper quartile and below the lower quartile). The variables are temperatures (27°C–46°C), ox bile concentrations of 0.5%–2%, pH 2–4.8, different carbon sources, ethanol concentrations 1, 6, and 12%, lactic acid concentrations 1, 6, and 12%, and glucose at 50 and 60%.

Further investigation of the strains among the *K. marxianus* species, revealed that there are significant differences between the two genetically different groups (*p* < 0.05, Wilcoxon rank sum test) (Figure 7).

Group I (G-nucleotide group) showed significantly better growth at elevated temperatures (42 and 46°C), at pH 3, on xylose and on phytic acid than Group II (A-nucleotide group). Group I also showed remarkably higher growth in presence of lactic acid, using a medium of yeast extract (1%), peptone (2%) and lactic acid at 1 and 6%. Group I showed higher tolerance toward osmotic stress compared to Group II. Group II on the other hand showed stronger growth on the sucrose and lactose based media, compared to Group I. Among the other two species (*S. cerevisiae* and *P. fermentans*) there were no significant differences in phenotypes found.

All isolates were also investigated for (i) invasiveness on YPD agar, (ii) resistance toward oxidative stress induced by H_2_O_2_ and (iii) formation of hyphae in different media (Table 3). The *K. unispora* showed low resistance toward oxidative stress, no invasiveness and no formation of hyphae. Within the three other species there were several strains (31% of *K. marxianus* strains, 40% of *S. cerevisiae* strains, and 33% of *P. fermentans* strains) showing hyphae formation in at least one of the tested media. The invasiveness was generally low in *K. marxianus* with only 10% of strains showing higher grading than 1 in invasiveness. The strains of the *S. cerevisiae* species were especially interesting by having either no invasiveness (70% of isolates) or very high invasiveness (30% of isolates). One strain of *S. cerevisiae* (CL2) and three strains of *K. marxianus* (AL1, AL4, CL5) showed high resistance toward hydrogen peroxide by having a distance of 5 mm or lower from the H_2_O_2_ disk and growth boarder.

**Table 3:** **Invasiveness of each isolate in YPD agar was assessed and graded from 0 (not invasive) to 4 (highly invasive), where “b” indicates more intense invasiveness at the colony border**.

**Species**	**Strain**	**H_2_O_2_ resistance (mm) 27°C**	**Invasive (0−4) 27°C**	**Hyphae (168 h)**				
				**YPD 27°C**	**YPD 37°C**	**YNB 27°C**	**YNB 37°C**	**RPMI 37°C**
*K. marxianus*	AL1	4	0	−	−	−	−	−
*K. marxianus*	AL2	9	0	−	−	−	−	−
*K. marxianus*	AL3	11	0	−	−	−	−	−
*K. marxianus*	AL4	4	0	−	−	−	−	−
*K. marxianus*	AL5	11	0	−	−	−	−	−
*K. marxianus*	BL1	10	1	−	−	−	−	−
*K. marxianus*	BL3	10	1	+	+	+	−	+
*K. marxianus*	BL4	14	0	−	−	−	+	−
*K. marxianus*	BL5	9	1	+	−	+	+	−
*K. marxianus*	BL6	10	0	−	+	−	−	−
*K. marxianus*	BL7	11	0	−	−	−	−	−
*K. marxianus*	BL8	11	0	−	−	−	−	−
*K. marxianus*	BL12	10	0	−	−	−	−	−
*K. marxianus*	BL13	12	0	−	−	−	−	−
*K. marxianus*	BL14	13	3	−	−	−	−	−
*K. marxianus*	CL5	4	2	+	+	+	+	+
*K. marxianus*	CL6	13	0	−	−	−	−	−
*K. marxianus*	DL2	13	0	−	−	−	−	−
*K. marxianus*	DL4	12	2 b	−	−	−	−	−
*K. marxianus*	DL5	11	0	−	−	−	−	−
*K. marxianus*	DL6	12	0	−	−	−	−	−
*K. marxianus*	DL10a	11	0	−	−	−	−	−
*K. marxianus*	DL10b	13	0	−	−	−	−	−
*K. marxianus*	DL11	13	1	−	−	−	−	−
*K. marxianus*	DL12	10	0	−	−	−	−	−
*K. marxianus*	TJY52	9	1	+	+	+	+	−
*K. marxianus*	TJY54	9	1	+	+	+	+	+
*K. marxianus*	TJY59	9	1	+	+	+	+	−
*K. marxianus*	TJY60	10	1	+	+	+	+	+
*S. cerevisiae*	BL9	17	3	+	−	+	−	+
*S. cerevisiae*	BL10	16	3	−	−	−	−	−
*S. cerevisiae*	BL11	9	0	−	−	−	−	−
*S. cerevisiae*	CL2	5	0	−	−	−	−	−
*S. cerevisiae*	CL3	14	0	−	−	−	−	−
*S. cerevisiae*	CL4	13	0	+	+	+	−	−
*S. cerevisiae*	DL3	17	0	−	+	−	+	+
*S. cerevisiae*	DL7	15	3 b	−	−	−	−	−
*S. cerevisiae*	TJY58	16	0	+	−	+	−	+
*S. cerevisiae*	TJY61	12	0	−	−	−	−	−
*P. fermentans*	BL2	11	2	+	+	+	−	+
*P. fermentans*	CL1	13	3	+	+	+	+	+
*P. fermentans*	CL7	11	1	−	−	−	−	−
*P. fermentans*	DL1	14	3	−	+	−	−	−
*P. fermentans*	DL8a	13	0	−	−	−	−	−
*P. fermentans*	DL8b	14	0	−	−	−	−	−
*P. fermentans*	DL9	15	1	−	−	−	−	−
*P. fermentans*	TJY50	9	1	−	−	−	−	−
*P. fermentans*	TJY53	14	1	−	−	−	−	−
*P. fermentans*	TJY55	13	1	−	−	−	−	−
*P. fermentans*	TJY56	13	1	+	+	+	+	+
*P. fermentans*	TJY57	14	1	−	−	−	−	−
*Kaz. unispora*	TJY51	12	0	−	n.d	−	n.d	n.d

*The hyphae formation, given as positive (+) or negative (−) was determined based on microscopic investigation after cultivation in the media YPD, YNB and RPMI respectively and at two different incubation temperatures. All experiments were carried out in duplicates and presented is the mean value. n.d indicates that no data was obtained, due to no growth at this temperature*.

The screening for phytate degradation after 48 h of incubation in a nutrient deficient medium revealed that only few isolates were able to degrade phytate under this condition, in particular isolates AL3 (43% IP_6_ degraded), BL8 (30% IP_6_ degraded), and BL1 (29% IP_6_ degraded). Isolates AL1, BL3, BL6, BL7, BL12, BL13, CL6, DL2, DL5, DL6, DL10b, DL11, and DL12 showed between 15 and 20% IP_6_ degradation. The remaining isolates showed no detectable levels of IP_6_ degradation. The reference strain *Pichia kudriavzevii* TY13 (Hellström et al., 2015) showed 93% IP_6_ degradation in this condition. The analysis of the isolates ability to release extracellular non-cell-bound phytase in an YNB+YE medium revealed no phytase activity in the supernatant from any of the investigated strains under tested conditions, phytase activity was however seen in the supernatant of the positive control references strain.

Selected strains were then subjected to determination of minimum inhibitory concentration (MIC) of selected antifungal agents (Table 4).

**Table 4:** **The minimum inhibitory concentration (MIC) of the antifungals fluconazole, clotrimazole, and amphotericine B are presented as mg/L needed for full inhibition**.

**Strain**	**Fluconazle (mg/L)**	**Clotrimazole (mg/L)**	**Amphotericin B (mg/L)**
BL3 (*K. marxianus*)	4	0.12	2
BL8 (*K. marxianus*)	8	0.5	8
DL4 (*K. marxianus*)	8	0.5	32^*^
TJY52 (*K. marxianus*)	8	0.03^**^	4
CL1 (*P. fermentans*)	64	0.03^**^	8
BL2 (*P. fermentans*)	128^*^	0.03^**^	16
CL2 (*S. cerevisiae*)	16	0.03^**^	2
BL9 (*S. cerevisiae*)	16	0.5	2
TJY51 (*K. unispora*)	128 ^*^	0.25	4

Additionally, a single asterisk (

* ) indicates that growth was observed at the highest tested concentration (i.e., MIC not determined), and double asterisk (

** *) indicates that no growth was observed even at the lowest tested concentration (i.e., MIC may be lower than the tested concentration)*.

The antifungal tolerance was varying between both species and strains. *P. fermentans* BL2 and *K. unispora* TJY51 showed resistance toward fluconazole (up to 128 mg/L), and *K. marxianus* DL4 showed resistance toward amphotericin B (up to 32 mg/L). Strains *K. marxianus* TJY52, *P. fermentans* CL1 and BL2, and *S. cerevisiae* CL2 were all inhibited already at the lowest tested concentration of clotrimazole (0.03 mg/L). Neither of the tested strains show resistance toward all antifungals, which is an important feature in order to allow external control of unwanted growth.

## Discussion

This is the first report on isolation, identification and characterization of yeast isolates from fermented goat milk of the Yaghnob Valley in Tajikistan. The yogurt contained a small variation of yeast species, dominated by the three species *K. marxianus, S. cerevisiae*, and *Pichia fermentans*. We isolated also one strain belonging to the species *K. unispora*. The same species that we identified in this work has also been isolated in other studies on fermented milk. In particular we observed that the yeast species composition of the Yaghnob fermentation is quite similar to that found in fermented Yak milk from the Tibetan plateau in China (Bai et al., 2010). However in other previous studies on traditional milk fermentations, a larger species diversity has generally been found as compared to our results. For example in *Amasi* made of cow milk (Gadaga et al., 2000) (20 different species, with the most predominant being *S. cerevisiae, Candida lusitaniae, C. colliculosa, S. dairensis*), in *Sameel* made of cow, goat, camel or sheep milk (Al-Otaibi, 2012) (36 different species, with the most prominent being *Candida lusitania, Cryptococcus laurentii, S. cerevisiae*), or in *Chal* made from camel milk (Yam et al., 2015) (35 species, with the most predominant being *Kluyveromyces lactis* and *K. marxianus* at 9% each). It seems as the predominant species, and the species variety, differs between different milk fermentations, probably both due to differences in raw material, physical factors (temperature, humidity etc.) and processing (the microflora of the people handling the fermentations, cleaning procedures, etc.).

Within the yeast species of the Yaghnob yogurt, the phenotypic analyses showed broad strain variation. All species presented remarkable differences in temperature tolerance, invasiveness, resistance to oxidative stress, hyphae formation, and inhibition by tested antifungals. It may be speculated that the broad strain variation within the three yeast species in the Yaghnob yogurt fermentation could be a phenotypic, and perhaps genotypic, adaptation restricted to the few species isolated in this fermentation niche.

Broad phenotypic strain variations within the *K. marxianus* species have previously been reported by, among others, Lane et al. (2011), where investigation of 13 strains from two European strain collections revealed variations in thermotolerance, tolerance to osmotic stress and to cell wall stress. The RFLP fingerprinting performed in this study revealed the presence of two groups (Group I and Group II) within the *K. marxianus* species. The strains belonging to Group II showed an RFLP pattern which to our knowledge has not been previously reported, and with a unique single nucleotide polymorphism in the ITS1-4 region compared to Group I. Since the ITS1-4 region is known to be well preserved, the nucleotide difference found in Group II may indicate other genetic variations between the two groups. From the phenotypic characterization of the strains, it became evident that there are also significant phenotypic differences between the two genetically different groups. Furthermore, Group I isolates, showing an RFLP pattern in accordance with those previously reported for *K. marxianus*, were isolated both from the original and maintained sample, while strains belonging to Group II, showing the novel RFLP pattern, was only isolated form the original sample. It may be speculated whether the strains of Group II constitutes a new species, and the genetic differences in those *K. marxianus* strains will be further investigated in future studies.

The strains of *S. cerevisiae* were in addition to ITS1-5.8S-ITS4 analyses, also assessed by microsatellites and used for creating a phylogenetic tree. Microsatellites are tandem repetitive DNA sequences of up to 10 nucleotides, which are spread throughout the genome and are inherited in a codominant matter (Pérez et al., 2001). Yeast microsatellite loci are reported to have a high degree of variability (Field et al., 1996). Previous articles described a set of microsatellite loci as successful in the discrimination between different *S. cerevisiae* strains (Field and Wills, 1998; Gallego et al., 1998; Pérez et al., 2001) enabling to discriminate beer, wine and bread strains from strains from other sources (Legras et al., 2005). Interestingly, all isolates from the Yaghnob Valley fermented milk clustered apart from previous isolates of *S. cerevisiae* collected from a wide variety of ecological niches, indicating that a separate evolution may have occurred in the geographically isolated area of the Yaghnob Valley. As observed for the *K. marxianus* strains, also the *S. cerevisiae* strains showed two different genetic backgrounds, based on ancestry analysis. One group, containing strains CL2, BL11, and DL7, originated from two ancestors, while the other group, consisting of strains CL3, DL3, CL4, BL10, BL9, TJY58, and TJY61, originated from three ancestors, two of them being common with the ancestor of the first group. The majority of *S. cerevisiae* strains could be the result of either convergent selection or, more likely, of clonal expansion. Still, as previously shown for strains isolated from fermenting beers and breads (Liti et al., 2009), all these strains bear a mosaic genome and were inferred to descend from two shared ancestors. Several strains in addition showed to descend from a third ancestor shared with strains isolated from wasp intestine. Furthermore, the phenotypic assessment of the *S. cerevisiae* strains revealed some variations in tolerances to low pH and high temperatures. In a study by Edwards-Ingram and co-workers (Edwards-Ingram et al., 2007), the comparison of the probiotic *S. boulardii* strains and other *S. cerevisiae* was done, and one of the phenotypic traits that appeared to separate the *S. boulardii* strains was the increased tolerance toward high temperatures and low pH. This led us to suggest that some of our isolated *S. cerevisiae* strains could in fact be *S. cerevisiae var. boulardii*. Especially the two strains BL9 and BL10, which show temperature tolerance up to 46°C and good growth at pH3, could according to the work by Edwards-Ingram et al. potentially belong to *S. cerevisiae var. boulardii*. As *S. boulardii* is a subtype of *S. cerevisiae* (Edwards-Ingram et al., 2004) they are difficult to separate based on the genomic work we performed in this study, hence further investigation of those strains genetic and phenotypic variations, as well as their potential probiotic effects need to be evaluated.

The fermented milk from the Yaghnob Valley is consumed without any prior sterilization step, meaning it contains viable cells when consumed. As several strains in this study show the ability to survive the conditions occurring in the intestinal tract (low pH, temperatures of 37°C and presence of ox bile), possible beneficial traits of those strains may be carried into the host. Other groups have investigated the probiotic potential of a strain of *K. marxianus* (BO399), presenting for example a positive effect on the immune response in CaCo-2 cell line (Maccaferri et al., 2012) and a positive effect on patients with irritable bowel syndrome (IBS) (Lisotti et al., 2013). The strains of this species isolated from the Yaghnob yogurt are therefore especially interesting for further studies of their possible probiotic properties.

One well-studied effect of yeast fermentation in cereal based foods is the degradation of the anti-nutrient phytate (IP_6_) and subsequent release of minerals (Fredrikson et al., 2002; Hellström et al., 2010) by phytase enzymes originating from the present microorganisms (Lopez et al., 2001; Reale et al., 2004; Nielsen et al., 2007). Although the strains in this work were isolated from a dairy fermentation, all strains were tested for the ability to degrade phytate under nutrient starved conditions. Several strains showed the ability to degrade phytate, although further investigations are needed in order to identify the optimal cultivation condition for an improved phytate degradation. Since degradation of phytic acid has shown to increase the mineral availability from cereal based foods (Sandberg et al., 1999; Lopez et al., 2001; Hurrell et al., 2002; Schlemmer et al., 2009), phytase positive strains may be industrially interesting not only in dairy fermentations, but also in cereal based fermentations. It may further be hypothesized that consuming viable phytase active yeasts, e.g., from the fermented Yaghnob milk, together with a cereal based meal may aid phytic acid degradation and subsequent mineral release inside the intestinal tract. Traits such as phytase activity, ethanol tolerance and lactic acid tolerance further indicate potential for use also in e.g., sourdough fermentations, where co-fermentation between yeast and lactic acid bacteria (LAB) occurs (Di Cagno et al., 2014). It is widely known that co-fermentation between yeasts and LAB takes place in many natural food fermentations, which is further supported by several previous studies (Narvhus and Gadaga, 2003; Al-Otaibi, 2012; Nyambane et al., 2014) where isolation of both of them has been done from the same fermentation sample. One interesting study by Plessas et al. (2008) investigated sourdough fermentations with *K. marxianus* together with the two LAB, *Lactobacillus delbrueckii* ssp. *bulgaricus* and *Lactobacillus helveticus*, revealing promising results such as prolonged shelf life, improved resistance to spoilage moulds and improved sensory qualities of the bread product. This indicates another interesting potential application for some of the strains isolated from the Yaghnob yogurt, especially since bacterial isolation from this same yogurt resulted in isolation of *Lactobacillus delbrueckii* and *Lactobacillus helveticus* as the two main species (data not published).

## Conclusions

This study present the first ever yeast isolation from fermented goat milk of the geographically isolated Yaghnob Valley. Genetic and phenotypic differences among strains were observed; (i) a single-nucleotide difference separating *K. marxianus* strains into two groups, (ii) *S. cerevisiae* strains phylogenetically clustering apart from a large set of previously isolated strains—the mosaic nature of these strains, together with the role of wasps gut as favoring sporulation and mating of *S. cerevisiae* (Stefanini et al., 2016)—suggests the gut as an unexplored niche for *S. cerevisiae*, (iii) phenotypic intra-species variations, e.g., ability to resist high temperatures, low pH and presence of ox bile, indicating their potential to survive the human gastro-intestinal tract.

## Author contributions

LQ was responsible for performing the experiments, analysing most of the data and for writing the manuscript. LQ, FS, and PM planned most of the experiments. IS and MS were responsible for handling and analysing the microsatellite data. GF was responsible for yeast isolations from original sample. TA, CDF, and DC were involved in supervision and discussions of the work. All authors were involved in revising the manuscript.

### Conflict of interest statement

The authors declare that the research was conducted in the absence of any commercial or financial relationships that could be construed as a potential conflict of interest.
